# Disulfidptosis-related gene in acute myocardial infarction and its diagnostic value and functions based on bioinformatics analysis and machine learning

**DOI:** 10.3389/fcvm.2025.1513342

**Published:** 2025-07-02

**Authors:** Liwen Chen, Jinru Wei, Guoxiong Deng, Guien Xu

**Affiliations:** ^1^ Department of Cardiology, The Fifth Affiliated Hospital of Guangxi Medical University, Nanning, Guangxi, China; ^2^ Department of Cardiology, The First People’s Hospital of Nanning, Nanning, Guangxi, China

**Keywords:** acute myocardial infarction, disulfidptosis, bioinformatics analysis, biomarker, ROC analysis

## Abstract

**Background:**

Acute myocardial infarction (AMI) is a major cause of morbidity and mortality. Disulfidptosis, a novel form of programmed cell death, has been largely unexplored in AMI. This study aims to identify disulfidptosis-related genes in AMI and assess their diagnostic potential using bioinformatics and machine learning.

**Methods:**

The microarray datasets GSE60993 and GSE61144, associated with Acute Myocardial Infarction (AMI), were obtained from the Gene Expression Omnibus (GEO) database. Differential disulfidptosis-associated genes were identified via differential expression analysis. The disulfidptosis related genes were collected from FerrDb V2 and the differentially expressed disulfidptosis related genes were utilized to construct a Protein-Protein Interaction (PPI) network. Key genes were identified utilizing a Protein-Protein Interaction (PPI) network and plugins available in Cytoscape. The key genes were used to detect potential biomarkers by receiver operating characteristic (ROC) analysis.Next, GO and KEGG analyses, as well as correlation analysis were performed on the key genes, and potential drug molecules targeting these genes were also analyzed. At the same time, key genes further screened by Support Vector Machine (SVM), Lasso regression, as well as random forest. By intersecting the results of the three, we ended up with hub genes for AMI. The expression of these key genes was verified using external dataset GSE61144.

**Results:**

A total of 16 differentially expressed disulfidptosis related genes were identified and these genes were mainly enriched in the pathways of “regulation of actin cytoskeleton organization”, “regulation of actin filament-based process”, “regulation of actin filament organization”, “cell cortex”, “cell leading edge”, “cadherin binding”, “actin filament bindin, and “D-glucose transmembrane transporter activity”. The top 10 hub genes ACTB, RAC1, IQGAP1, FLNB, MYL6, ABI2, DBN1, PRDX1, SLC2A1 and SLC2A3 were identified from the PPI network. Further screening using Support Vector Machine (SVM), Lasso regression and random forest, and intersecting the results of these analyses, led to the identification of DBN1, RAC1, and SLC2A3 as final hub genes in AMI. While the final key genes DBN1 and SLC2A3 were significantly differentially expressed in external dataset GSE61144 with AUC ≥ 0.7.

**Conclusion:**

In this study, we identified differentially expressed disulfidptosis related genes in blood samples from AMI patients using existing datasets. The research delved into the expression patterns and molecular mechanisms of differentially expressed disulfidptosis related genes in AMI, offering a foundation for precise AMI diagnosis and the identification of novel therapeutic targets.

## 1 Introduction

Cardiovascular diseases remained a major cause of premature mortality and increased healthcare costs (1, 2). According to the 2019 Global Burden of Cardiovascular Diseases and Risk Factors Study, cardiovascular diseases exhibited a rising trend globally. The total number of cases nearly doubling from 271 million in 1990 to 523 million in 2019, and the number of deaths increasing from 12.1 million in 1990 to 18.6 million in 2019. Cardiovascular diseases continued to be a leading cause of global disease burden. Among these, ischemic heart disease was the most prominent, with 9.74 million deaths attributed to it in 2019 (2). Acute myocardial infarction, a subtype of ischemic heart disease, which was characterized by a rapid onset, high incidence, and mortality rates (3), with a poor prognosis. It was identified as one of the leading causes of mortality and disability among middle-aged and elderly individuals worldwide (4).

Acute myocardial infarction was typically caused by the rupture or erosion of coronary atherosclerotic plaques, platelet activation, and subsequent coronary thrombotic occlusion, which led to myocardial ischemia, injury, and necrosis. Following myocardial infarction, various cellular signaling pathways were activated. Oxidative stress and tissue death, particularly apoptosis and necrosis of myocardial cells, triggered an inflammatory response. Immune cells infiltrated the infarcted myocardial region and released inflammatory factors. The inflammatory response, combined with pathological myocardial hypertrophy and reactive fibrosis, ultimately led to cardiac remodeling and heart failure (5). Persistent coronary occlusion or ischemia-reperfusion injury stimulated extensive myocardial cell death in the ischemic region (6, 7), with myocardial cells undergoing apoptosis (8, 9), necrosis (9–12), and autophagy (13–15), resulting in significant irreversible loss of myocardial cells. Therefore, myocardial cell death played a critical role in the pathogenesis and progression of myocardial infarction.

Due to the limited regenerative and repair potential of myocardial cells, dead cells could not be replaced by viable myocardial cells (16). It was reported that increased myocardial cell survival and reduced apoptosis could enhance myocardial functional recovery and promote left ventricular functional restoration by decreasing programmed myocardial cell death (17). Thus, timely intervention in myocardial cell death was of significant importance for improving the prognosis of myocardial infarction.Immediate restoration of coronary blood flow early and effective reperfusion therapy was identified as the primary treatment goals for acute myocardial infarction (18). Reperfusion therapy included thrombolysis and direct percutaneous coronary intervention (PCI). Studies indicated that PCI was the most effective reperfusion therapy for improving clinical outcomes in patients with ST-segment elevation myocardial infarction (19). Currently, chest pain characteristics, electrocardiogram results, and high-sensitivity cardiac troponin are commonly used diagnostic criteria for acute myocardial infarction. However, high-sensitivity cardiac troponin still has limitations in differentiating early myocardial infarction, mild myocardial injury, aortic dissection, pulmonary embolism, or chronic coronary syndrome (20). Identifying novel biomarkers and elucidating cell death mechanisms are crucial for early diagnosis and treatment.

Cell death is a fundamental feature of life and death (21). It plays a crucial role in normal biological processes such as embryonic development and postnatal homeostasis. When cell death is excessive, reduced, or misplaced, it can play a major role in human diseases, including cardiovascular diseases, diabetes, and cancer (22). During acute myocardial infarction, extensive myocardial cell death occurs in the infarcted region, with apoptosis being one of the main forms of myocardial cell death during myocardial infarction (23). Research has shown that pro-apoptotic proteins such as Bax are overexpressed in ischemic myocardial tissue, and inhibiting Bax activation can reduce apoptosis, thereby mitigating ischemia-reperfusion injury in myocardial infarction (24). Overexpression of the cardiac-specific anti-apoptotic protein Bcl-2 significantly alleviated myocardial cell apoptosis and infarct size following ischemia-reperfusion injury (25). Current research indicates that autophagy plays distinct roles at different stages of acute myocardial infarction. During the acute ischemic phase of acute myocardial infarction, insufficient ATP production in myocardial cells can induce autophagy. Autophagic degradation can release energy substrates such as free fatty acids and amino acids, alleviating the energy crisis and promoting myocardial cell survival (26). Simultaneously, autophagy can facilitate mitochondrial renewal by clearing dysfunctional mitochondria and preventing the release of cysteine-containing aspartic protease (caspase-3), thus reducing apoptosis and protecting the myocardium (27). Conversely, during the ischemia-reperfusion phase of acute myocardial infarction, excessive activation of autophagy can lead to autophagic cell death, exacerbating myocardial cell damage. Studies have reported that inhibiting autophagy by reducing Beclin1 expression through RNA interference (RNAi) or uric acid treatment can protect myocardial cells during ischemia-reperfusion (28). Ferroptosis, a form of iron-dependent cell death characterized by oxidative damage to the cell membrane, has been confirmed to occur in both myocardial and non-myocardial cells during myocardial ischemia-reperfusion (29). Ferroptosis inhibitors, such as ferrostatin-1, have been shown to prevent myocardial cell death and reduce infarct size in both cardiac transplantation and traditional coronary ligation ischemia-reperfusion models (30). During myocardial ischemia-reperfusion or non-reperfusion myocardial infarction, the expression of inflammasome components and activation of caspase-1 are upregulated. Activated caspase-1 promotes the maturation of inflammatory cytokines and induces pyroptosis (31). Conversely, the absence of adapter protein (ASC) and caspase-1 in the inflammasome reduces inflammation and mitigates myocardial infarction progression (32). The activation of the NLRP3/ASC/caspase-1 pathway and high levels of interleukin-1β (IL-1β) can induce pyroptosis (33). Studies have shown that silencing caspase-1 can inhibit the activation of the NLRP3/ASC/caspase-1 axis, reducing myocardial functional impairment caused by ischemia-reperfusion injury (34). Thus, pyroptosis plays a role in the myocardial infarction process.

**Figure 1:**
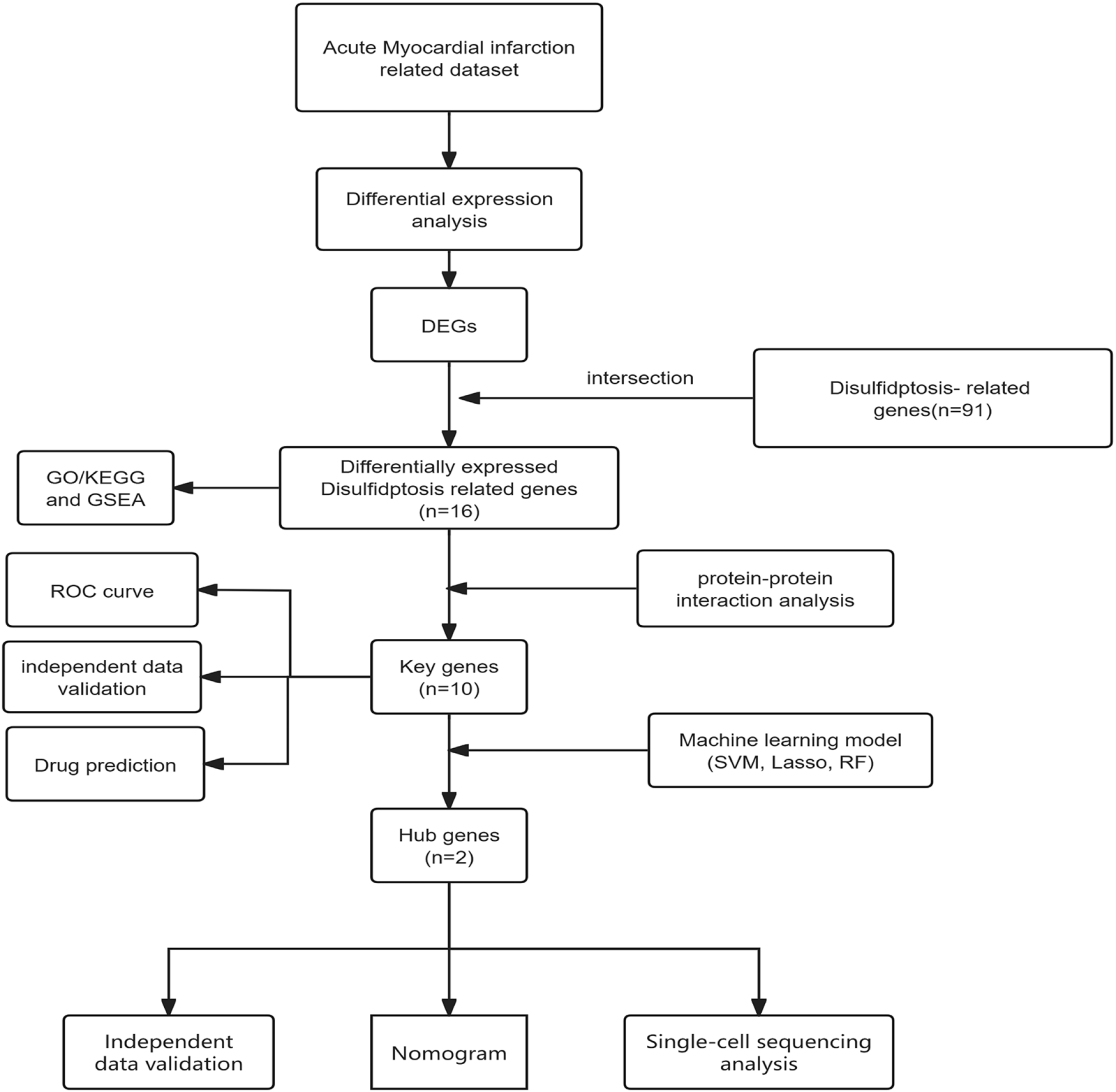
Diagram showing the sequence of steps conducted in this research.

Given that various forms of myocardial cell death are involved in acute myocardial infarction, studying the molecular mechanisms of myocardial cell death and exploring new biomarkers hold significant importance for the diagnosis and treatment of myocardial infarction. Disulfidptosis,a novel form of programmed cell death reported for the first time in March 2023, occurs under glucose-deprived conditions. High expression of cystine transporter solute carrier family 7 member 11 (SLC7A11) leads to rapid depletion of intracellular NADPH, resulting in significant accumulation of disulfides and rapid cell death. This form of cell death cannot be prevented by various cell death inhibitors, including ferroptosis inhibitors, apoptosis inhibitors, and necroptosis inhibitors, but can be completely inhibited by disulfide bond reducing agents such as dithiothreitol (DTT) and β-mercaptoethanol.Elevated intracellular cystine levels induce cytoskeletal disorganization through NADPH-dependent redox imbalance, triggering pathological disulfide crosslinking in actin-associated proteins. This redox perturbation leads to rapid collapse of branched actin networks, characterized by dissolution of lamellipodial structures and subsequent cell contraction, through mechanisms independent of reactive oxygen species. Functional genomic analyses identified the WAVE regulatory complex (WRC), particularly its core components NCKAP1 and RAC1, as essential mediators connecting actin polymerization dynamics to disulfide stress-induced cell death (35). Although no direct studies have explored the relationship between disulfidptosis and acute myocardial infarction (AMI), substantial evidence indicates that oxidative stress plays a pivotal role in AMI pathogenesis. As mentioned before, various cell death mechanisms are involved in myocardial infarction, and thus, disulfide cell death may also be associated with acute myocardial infarction.During myocardial ischemia/reperfusion injury, mitochondrial dysfunction and elevated oxidative stress contribute to increased cystine accumulation and compromised glutathione (GSH) synthesis—factors that may collectively create a cellular environment permissive for disulfidptosis. Furthermore, cardiomyocytes are characterized by an extensive actin-based cytoskeletal network, which is particularly vulnerable to disturbances in protein folding and aberrant disulfide bond formation.Given this established link, we hypothesize that disulfidptosis, as a form of programmed cell death involving oxidative stress, could also contribute significantly to the development and progression of AMI.Therefore, identifying and analyzing disulfidptosis-related genes in acute myocardial infarction through bioinformatics techniques, which hold promise for providing new insights into the diagnosis and treatment of acute myocardial infarction. The workflow for the specific analysis is illustrated in Figure 1.

## 2 Materials and methods

### 2.1 Collect and sort data

The following raw datasets were retrieved from the GEO database (https://www.ncbi.nlm.nih.gov/geo/) using the “GEOquery” R package (36): GSE60993(mRNA), GSE61144(mRNA). GSE61144 is derived from the GPL6106 platform (Sentrix Human-6 v2 Expression BeadChip), while GSE60993 originates from the GPL6884 platform (Illumina HumanWG-6 v3.0 Expression BeadChip). These datasets include gene expression data from both Acute myocardial infarction (AMI) patients and control groups. Specifically, the GSE60993 dataset comprised 17 AMI human peripheral blood samples and 7 control samples; the GSE61144 dataset included 14 AMI samples and 10 control samples. Table 1 presents the details of the two datasets sourced from the GEO database.

**Table 1:** The information of the 2 microarray datasets obtained from the GEO database.

Data source	Organism	Platform	Year	Sample source	Sample size (AMI/CON)	Detected RNA type
GSE60993	Homo Sapiens	GPL6884	2015	Blood	17/7	mRNA
GSE61144	Homo Sapiens	GPL6106	2015	Blood	14/10	mRNA

### 2.2 Identification of genes with differential expression related to acute myocardial infarction and disulfidptosis

Differential gene analysis was conducted utilizing the R package “limma” (37). Genes were considered differentially expressed (DEGs) if the |log2 fold change (FC)| was greater than 0 and the *P*-value was less than 0.05 (38, 39). The results of the differential expression analysis were visualized using volcano plots and heatmaps. A total of 91 regulatory factors, including drivers, suppressors, and unclassfied, were retrieved from the FerrDB V2 database (Supplementary File S1). Differentially expressed genes (DEGs) from GSE60993 were intersected with the genes obtained from FerrDB V2 to identify DEGs associated with disulfidptosis. Additionally, Gene Ontology (GO) enrichment analysis and Kyoto Encyclopedia of Genes and Genomes (KEGG) pathway analysis were carried out with the “clusterProfiler” package (40) in R to further elucidate the biological functions of the DEGs associated with disulfidptosis (DRGs).

### 2.3 Development of the protein-protein interaction network and identification of key genes

To elucidate the interaction mechanism between DEGs associated with disulfidptosis(DRGs) in AMI, a protein-protein interaction (PPI) network was constructed using the STRING database (version 10.0)6. This network was visualized with Cytoscape software (version 3.7.1). The CytoHubba plug-in within Cytoscape was employed to identify the top 10 key genes with the highest connectivity in the PPI network using the Closeness Centrality algorithm.

### 2.4 Support vector machine, lasso regression and the development of the random forest model

Support Vector Machine (SVM), Lasso regression, and the Random Forest model were employed to further screen the hub genes among the top 10 key genes in AMI obtained above. R version 4.2.3 was used to analyze all three machine learning methods. SVM is a powerful algorithm that finds an optimal hyperplane to separate different classes. Lasso regression imposes a penalty on the model coefficients to perform variable selection and regularization. The Random Forest model, which aggregates multiple decision trees, was used to further refine gene selection. The selection of these methods was driven by their distinct advantages in genomic feature selection: LASSO regression utilized L1 regularization to mitigate multicollinearity in high-dimensional datasets, producing sparse solutions by setting the coefficients of non-predictive variables to zero, with the optimal regularization parameter (*λ*) identified through 10-fold cross-validation using cv.glmnet, and significant features defined as those retaining non-zero coefficients at *λ*min.The optimal regularization parameter (*λ*.min = 0.0318) eliminated 50% of coefficients through L1 regularization (*α*=1), retaining 40 genes with non-zero coefficients.For the SVM approach, an RBF-kernel SVM was integrated with RFE to manage non-linear relationships in small-sample datasets, where feature subsets ranging from 2 to 40 variables were evaluated via 10-fold cross-validation, with the optimal subset size determined by minimizing the root mean squared error (RMSE); the random forest model adopted an ensemble strategy involving 500 decision trees, with mtry set to 2 (optimized via grid search), and variable importance assessed based on the mean decrease in node impurity across 10-fold cross-validation iterations. Among candidate mtry values (2, 6, 10), the configuration with mtry = 2 achieved peak classification accuracy (86.7%). All analyses were performed in R (version 4.2.3) with fixed random seeds [set. seed(123)] to ensure reproducibility, utilizing the glmnet, caret, and randomForest packages for model implementation. Genes identified by these three methods were intersected to determine the hub genes associated with AMI.

### 2.5 Construction of nomograms

R version 4.2.3 was used to analyze the regression line chart prediction model. A nomogram is based on a multivariate regression model. Each gene is assigned a score based on its contribution to acute myocardial infarction, and these scores are then summed to estimate the probability of developing the disease. Nomograms are becoming increasingly popular in clinical settings due to their ability to convert complex regression equations into straightforward visual representations. The scales on the nomogram enable clinicians to easily evaluate a patient's risk of acute myocardial infarction.

### 2.6 Additional validation of key genes was performed using the external dataset GSE61144

The results from Support Vector Machine (SVM), Lasso regression, and the Random Forest model analyses were compared to identify hub genes associated with acute myocardial infarction. Integrating these hub genes into nomograms enhances the clarity of predictions and improves the model's interpretability. The diagnostic utility of these top 10 key genes was assessed using the dataset GSE61144. The GSE61144 dataset included 14 AMI human peripheral blood samples and 10 control samples. ROC curves were generated for each key gene to evaluate its diagnostic performance, and the differential expression of key genes across the external dataset was analyzed.

### 2.7 Drug prediction (DSigDB)

To identify potential drugs targeting key genes associated with acute myocardial infarction,predictive analysis was conducted using the DSigDB database using Enrichr. And the top 5 scores were selected as candidate drugs.

### 2.8 Single cell sequencing analysis

Single-cell RNA sequencing data were processed utilizing the Seurat and SingleR computational frameworks. Initial quality control involved exclusion of cells with gene counts below 300 or exceeding 7,000, mitochondrial gene content surpassing 10%. Gene expression profiles underwent normalization via the Seurat-integrated normalization algorithm. Principal component analysis (PCA) was conducted on 2,000 highly variable genes, with the top 10 principal components retained for downstream analysis. Cellular subpopulations were delineated through unsupervised clustering (resolution = 0.4) by constructing neighbor graphs and applying Uniform Manifold Approximation and Projection (UMAP) for dimensionality reduction. Cluster-specific marker genes were identified using threshold criteria of adjusted *P* < 0.05 and min.pct = 0.25. Cell type annotation was performed through reference-based classification with SingleR. Principal component significance was validated via JackStraw permutation testing, retaining components demonstrating statistically significant deviation from null distributions. Differential expression patterns of ten key gengs across cell subsets were visualized through violin plots.

## 3 Results

### 3.1 Identification of genes with differential expression and variations in DRGs in acute myocardial infarction

**Figure 2:**
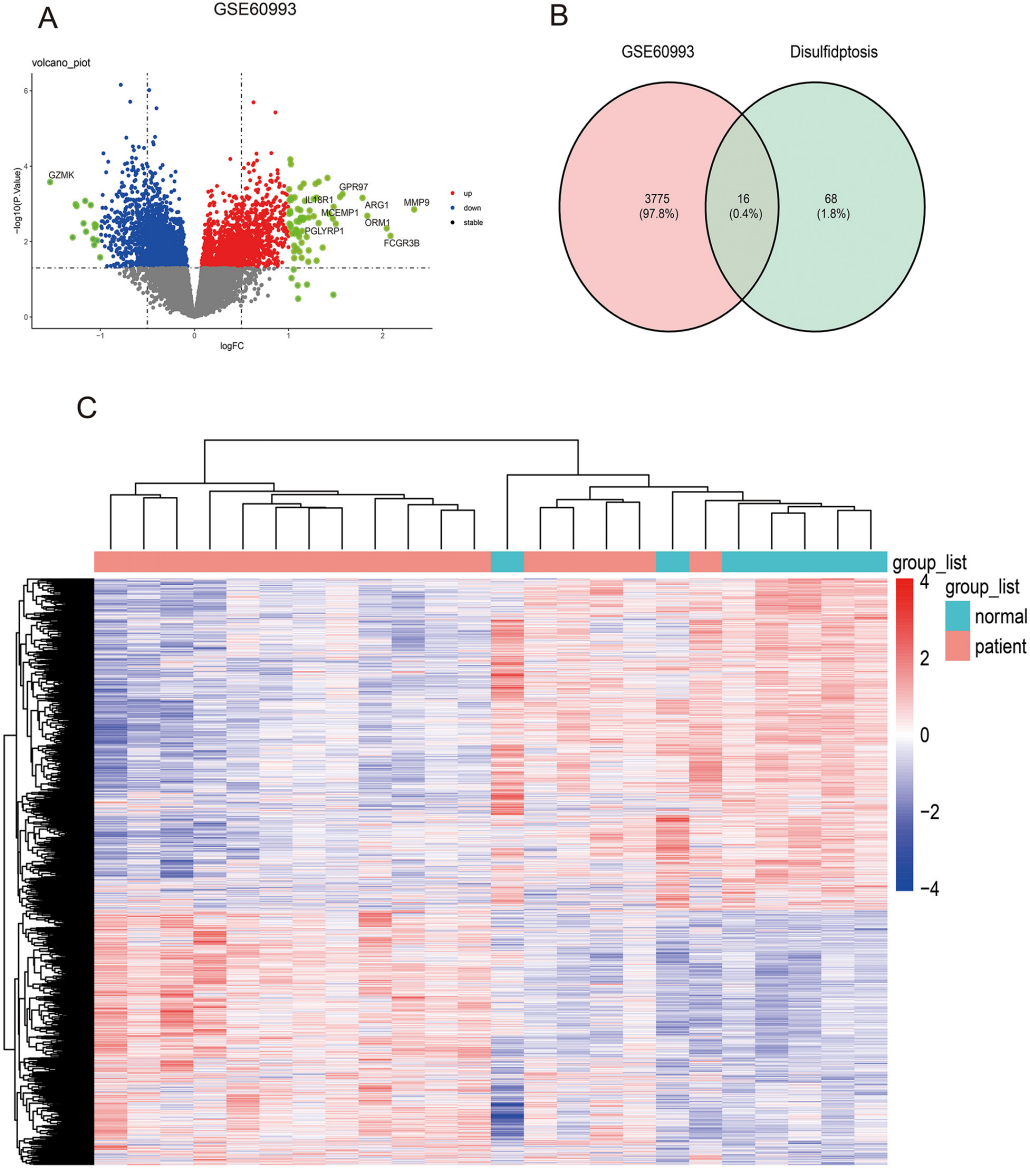
Identifying differentially expressed genes in dataset GSE60993 and differentially expressed DRGs. **(A)** The volcano plot of GSE60993. **(B)** Ven diagram of DEGs in GSE60993 and disulfidptosis-related genes. **(C)** The heatmap plot of GSE60993.

The AMI-related datasets GSE60993 was retrieved from the Gene Expression Omnibus (GEO) database. Using the aforementioned screening criteria (*P*-value < 0.05 and | logFC| > 0), differentially expressed genes (DEGs) were identified in the dataset. Specifically, GSE60993 yielded 3,791 DEGs, with 1,648 upregulated and 2,143 downregulated genes. Detailed results of the differential expression analysis are provided in Supplementary File S2. Table 2 lists the top 5 upregulated and top 5 downregulated DEGs for the dataset. The volcano plots illustrating these DEGs for GSE60993 is presented in Figure 2A. To identify DEGs and differentially expressed disulfidptosis-related genes (DRGs), an online Venn tool was employed, as shown in Figure 2B. This analysis identified 16 differentially expressed DRGs in GSE60993, as detailed in Supplementary File S3. Heatmaps of the differentially expressed genes for GSE60993 is displayed in Figures 2C.

**Table 2:** The top 5 upregulated DEGs and the top 5 downregulated DEGs in GSE60993.

Dataset	Type	DEG	Expression	Log2 FC	1. P. Value
GSE60993	mRNA	MMP9	up	2.3355267	1.405589e-03
GSE60993	mRNA	FCGR3B	up	2.0845665	1.405589e-03
GSE60993	mRNA	ORM1	up	2.0438146	4.444007e-03
GSE60993	mRNA	MCEMP1	up	1.8375565	2.078802e-03
GSE60993	mRNA	ARG1	up	1.7866877	6.930997e-04
GSE60993	mRNA	GZMK	down	−1.5337640	2.639576e-04
GSE60993	mRNA	CLC	down	−1.2937880	7.744503e-03
GSE60993	mRNA	HLA-DQA1	down	−1.2629242	1.023522e-03
GSE60993	mRNA	KLRB1	down	−1.2526464	1.139646e-03
GSE60993	mRNA	KLRG1	down	−1.1782455	3.288638e-03

Note: log2 FC > 0 indicates upregulation, while log2 FC < 0 denotes downregulation.

### 3.2 Enrichment analysis of genes associated with disulfidptosis that show differential expression

**Figure 3:**
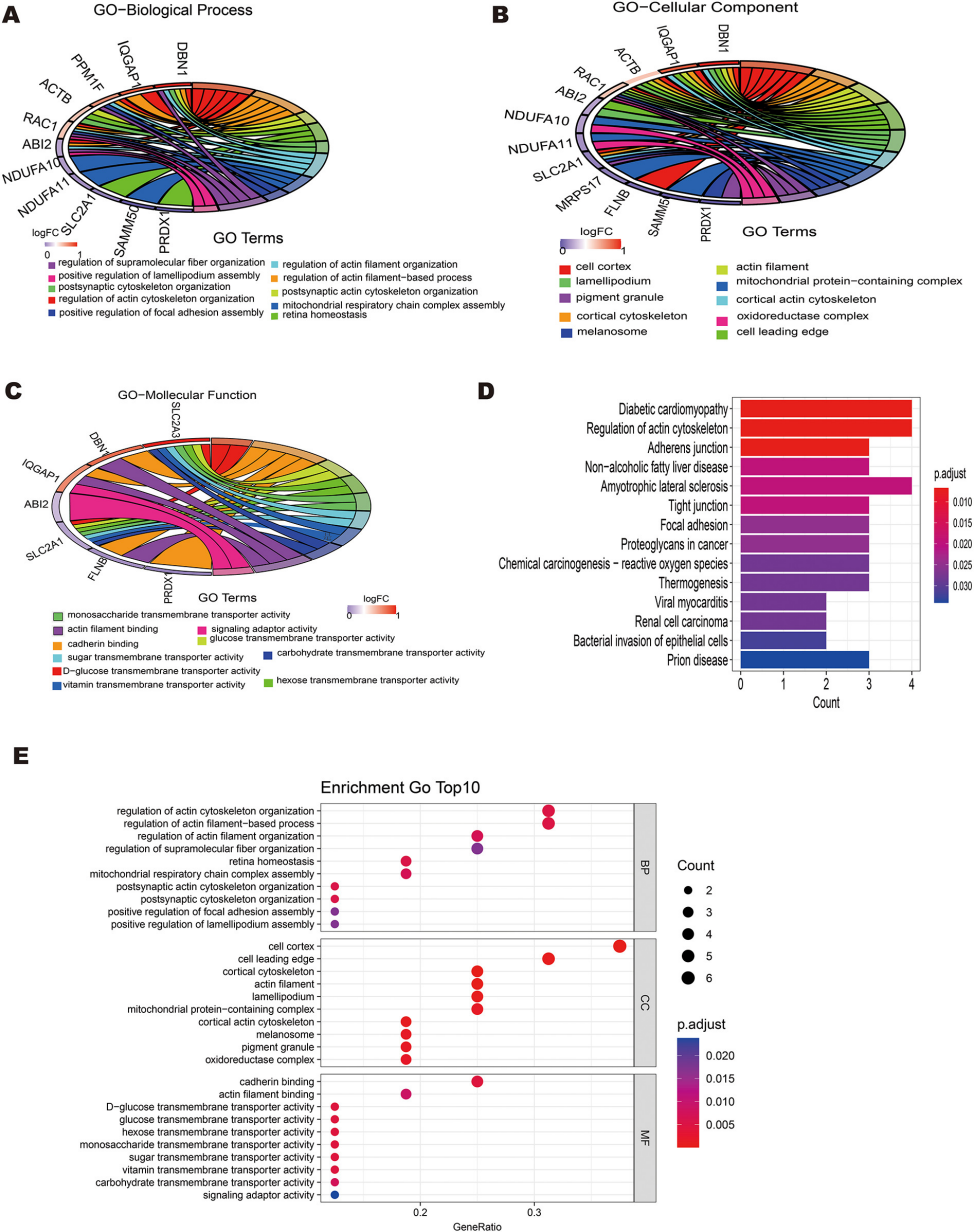
Enrichment analysis of 16 differentially expressed disulfidptosis-related genes(DRGs). **(A)** Circle plot showing the top 10 biological process pathway enrichments. **(B)** Circle plot displaying the top 10 cellular component pathway enrichments. **(C)** -Circle plot displaying the top 10 molecular function pathway enrichments.**(E)** Bar plot showing the top 10 KEGG pathway enrichments.

Gene ontology (GO) enrichment analysis and Kyoto Encyclopedia of Genes and Genomes (KEGG) pathway analysis were also performed using the “clusterProfiler” package in R to further investigate the biological roles of differentially expressed DRGs. The 16 differentially expressed DRGs in GSE60993 were analyzed using using the “clusterProfiler” package in R for GO annotation and KEGG pathway enrichment. Figures 3A–E display the top 10 enriched GO terms and KEGG pathways. For GO biological process (BP) analysis, the differentially expressed DRGs were primarily associated with terms such as “regulation of actin cytoskeleton organization,” “regulation of actin filamentbased process,”“ postsynaptic actin cytoskeleton organization,”“ retina homeostasis” and“postsynaptic cytoskeleton organization” (Figure 3A). In the GO cellular component (CC) analysis, the top 5 enriched terms were “cell cortex,” “cell leading edge,”“cortical cytoskeleton,”“actin filament,”and“lamellipodium”(Figure 3B). For GO molecular function (MF) analysis, the most significantly enriched terms included“cadherin binding,” “actin filament binding,”“D-glucose transmembrane transporter activity,” “glucose transmembrane transporter activity,”“hexose transmembrane transporter activity”and“monosaccharide transmembrane transporter activity” (Figure 3C). The results of this three-part GO enrichment analysis are presented in the graph (Figure 3D), showing the significance of each enrichment entry. Additionally, the KEGG analysis highlighted significant pathways such as “Diabetic cardiomyopathy,” “Regulation of actin cytoskeleton,”“Adherens junction,” “Non-alcoholic fatty liver disease,” and “Amyotrophic lateral sclerosis”(Figure 3E).

### 3.3 Development of a protein-protein interaction network and identification of key genes in acute myocardial infarction

**Figure 4:**
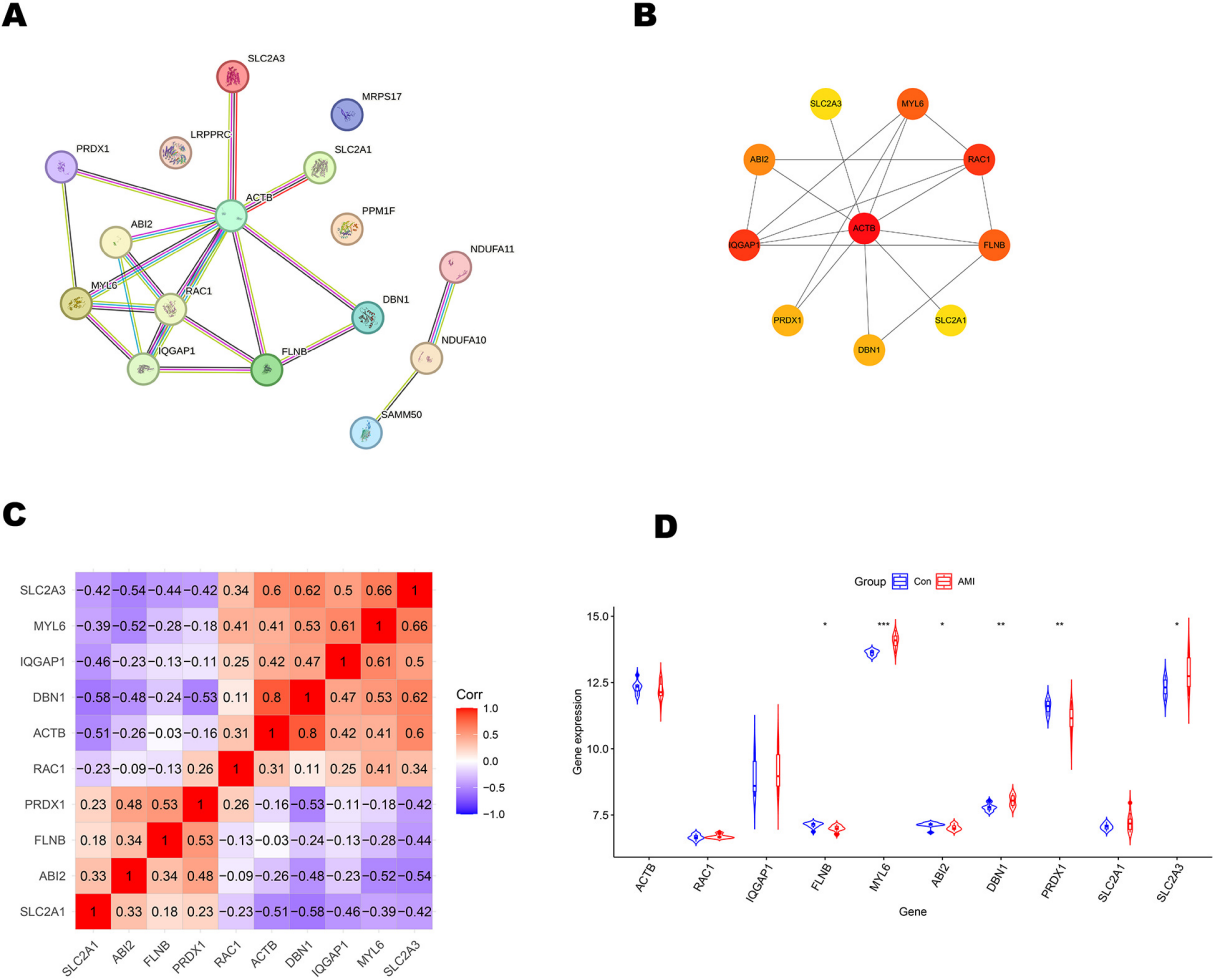
Primary screening and analysis of genes linked to disulfidptosis. **(A)** Network of protein-protein interactions. **(B)** The top 10 key genes as predicted by the Closeness algorithm. **(C)** In correlation analysis of 10 key genes, positive correlations are indicated by the color red, while negative correlations are denoted by the color blue. **(D)** Expression patterns of key genes in external dataset GSE61144, *P < 0.05; **P < 0.01; ***P < 0.001.

**Figure 5:**
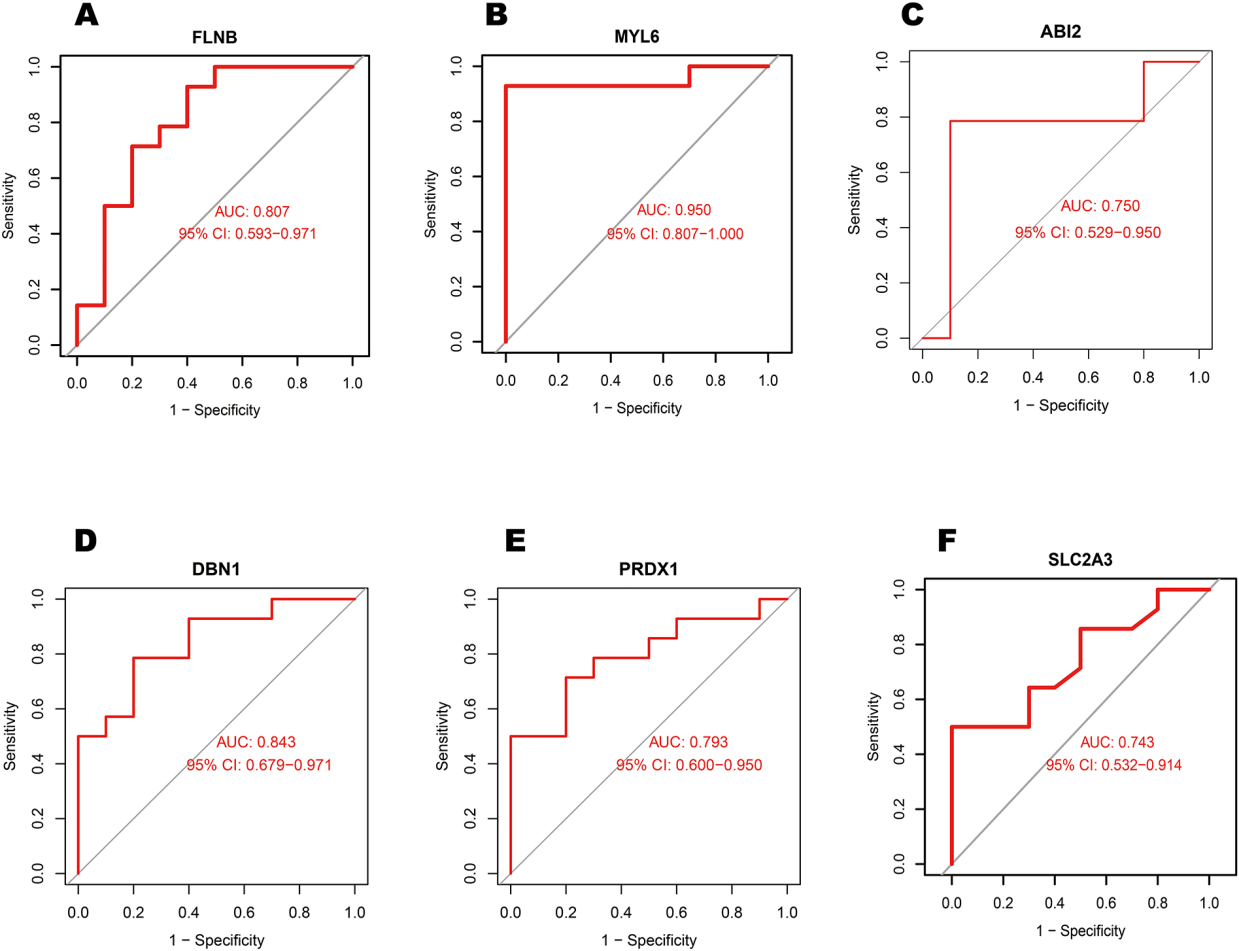
Analysis of the top 6 key genes AUC values in external dataset GSE61144 using receiver operating characteristic curves. **(A)** FLNB, **(B)** MYL6, **(C)** ABI2, **(D)** DBN1, **(E)** PRDX1, and **(F)** SLC2A3.

The 16 differentially expressed DRGs were analyzed using the String database to construct a protein-protein interaction (PPI) network. Applying the default parameters of the String database, a PPI network comprising 16 nodes and 20 edges was generated (Figure 4A). Subsequently, the top 10 key genes (ACTB, RAC1, IQGAP1, FLNB, MYL6, ABI2, DBN1, PRDX1, SLC2A1, and SLC2A3) were identified using the Closeness algorithm via the Cytohubba plugin in Cytoscape (Figure 4B). Further pathway enrichment analysis was conducted using the KOBAS (version 3.0) online tool. This analysis revealed that IQGAP1, FLNB, and ACTB are involved in the “Proteoglycans in cancer” pathway, IQGAP1, ABI2, and ACTB are associated with the “Regulation of actin cytoskelet”, IQGAP1 and ACTB are linked to the “Adherens junction”. The 10 key genes exhibited strong correlations, with DBN1 showing a notable positive association with ACTB (coefficient = 0.80), whereas DBN1 had a negative relationship with SLC2A1 (coefficient = −0.58) (Figure 4C). The expression of 10 key genes was further validated using the external dataset GSE61144 (Figure 4D). Among these genes, RAC1, IQGAP1, MYL6, DBN1, SLC2A1 and SLC2A3 were significantly upregulated in AMI patients compared to healthy controls. Conversely, ACTB, FLNB, ABI2 and PRDX1 were found to be downregulated in AMI patients. Using the raw expression data from GSE61144, ROC analysis was conducted on 10 key genes (ACTB, RAC1, IQGAP1, FLNB, MYL6, ABI2, DBN1, PRDX1, SLC2A1, and SLC2A3). The AUC values for these genes were calculated, and all were found to exceed 0.6. Specifically, 6 of these key genes demonstrated AUC values greater than 0.7, identifying them as potential biomarkers for AMI: FLNB(AUC = 0.807), MYL6 (AUC = 0.950), ABI2(AUC = 0.750), DBN1 (AUC = 0.843), PRDX1 (AUC = 0.793), SLC2A3 (AUC = 0.743). Figures 5A–F illustrate the ROC analysis results for the top 6 key genes with the highest AUC values. The ROC results for the remaining 4 key genes are provided in Supplementary Figure S1. Additionally, we used the DSigDB database to predict drugs that interact with 10 key genes and identified top 10 drugs targeting them (Table 3).

**Table 3:** The top 5 drugs prediction targeting key genes.

Term	1. P. Value	Odds ratio	Combined score	Genes
pentobarbital CTD 00006484	2.69E-05	356.71	3,753.85	SLC2A1; RAC1
CYTOCHALASIN CTD 00005746	1.11E-04	166.33	1,515.06	SLC2A1; SLC2A3
Benzethonium chloride HL60 DOWN	1.40E-04	146.73	1,301.61	SLC2A1; SLC2A3
quercetin CTD 00006679	1.87E-04	12.469	107.02	PRDX1;SLC2A1;FLNB;SLC2A3;IQGAP1;ACTB;DBN1
Uranium acetate CTD 00000229	2.00E-04	35.26	300.45	SLC2A3; RAC1; ACTB

### 3.4 Machine learning-based identification of a disulfidptosis signature

**Figure 6:**
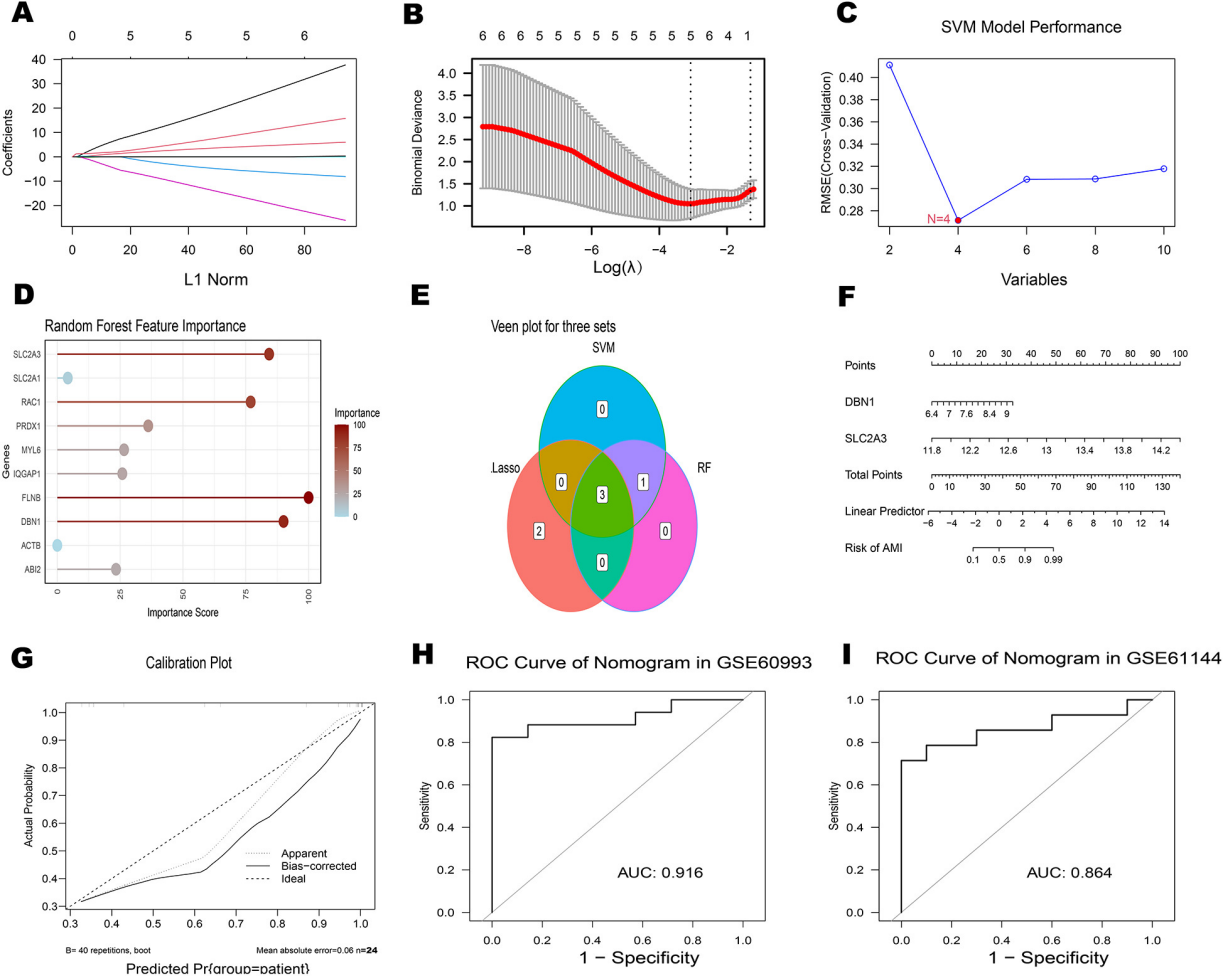
Identification of hub genes associated with AMI **(A)** plot of coefficient distribution for LASSO regression **(B)** cross-test maps of penalty terms. **(C)** SVM performance graph. **(D)** Random Forest feature importance plot. **(E)** The Venn diagram illustrates the overlap of candidate genes identified by three algorithms: LASSO, SVM and RF. **(F-I)** Assessment of ROC curves for the diagnostic performance of the 2-hub gene construct and nomogram in two datasets.

Using 10 key genes, we applied Support Vector Machine (SVM), Lasso regression, and the development of the Random Forest algorithms to identify potential genes and develop a disulfidptosis-related signature (Figures 6A–E). This analysis led to the identification of 2 disulfidptosis-associated feature genes as hub genes: DBN1 and SLC2A3. To assess the diagnostic potential of each feature gene for Acute myocardial infarction (AMI), a nomogram model was created as a diagnostic tool for AMI (Figures 6F,G). The AUC values of the ROC curves for the diagnostic performance of this nomogram in the GSE60993 and GSE61144 datasets were 0.916 and 0.864, respectively. (Figures 6H,I).

### 3.5 Additional validation of hub genes using the external dataset GSE61144

The expression levels of 2 hub genes were further examined in the external dataset GSE61144 (Figures 5A,F). Compared to healthy controls, DBN1 were significantly upregulated in AMI patients. Conversely, FLNB was found to be downregulated in AMI patients. The ROC curve analysis yielded AUC values of 0.743 for SLC2A3, 0.84 for DBN1,0.60 for RAC1 in GSE61144. The area under the ROC curve in the external validation dataset exceeded 0.6, demonstrating the effective diagnostic performance of the hub genes associated with AMI. As a result, only 2 genes (SLC2A3 and DBN1) are identified as the final hub genes.

### 3.6 Single-cell analysis reveals key genes-SMCs-Ec interactions and immune crosstalk in AMI

**Figure 7:**
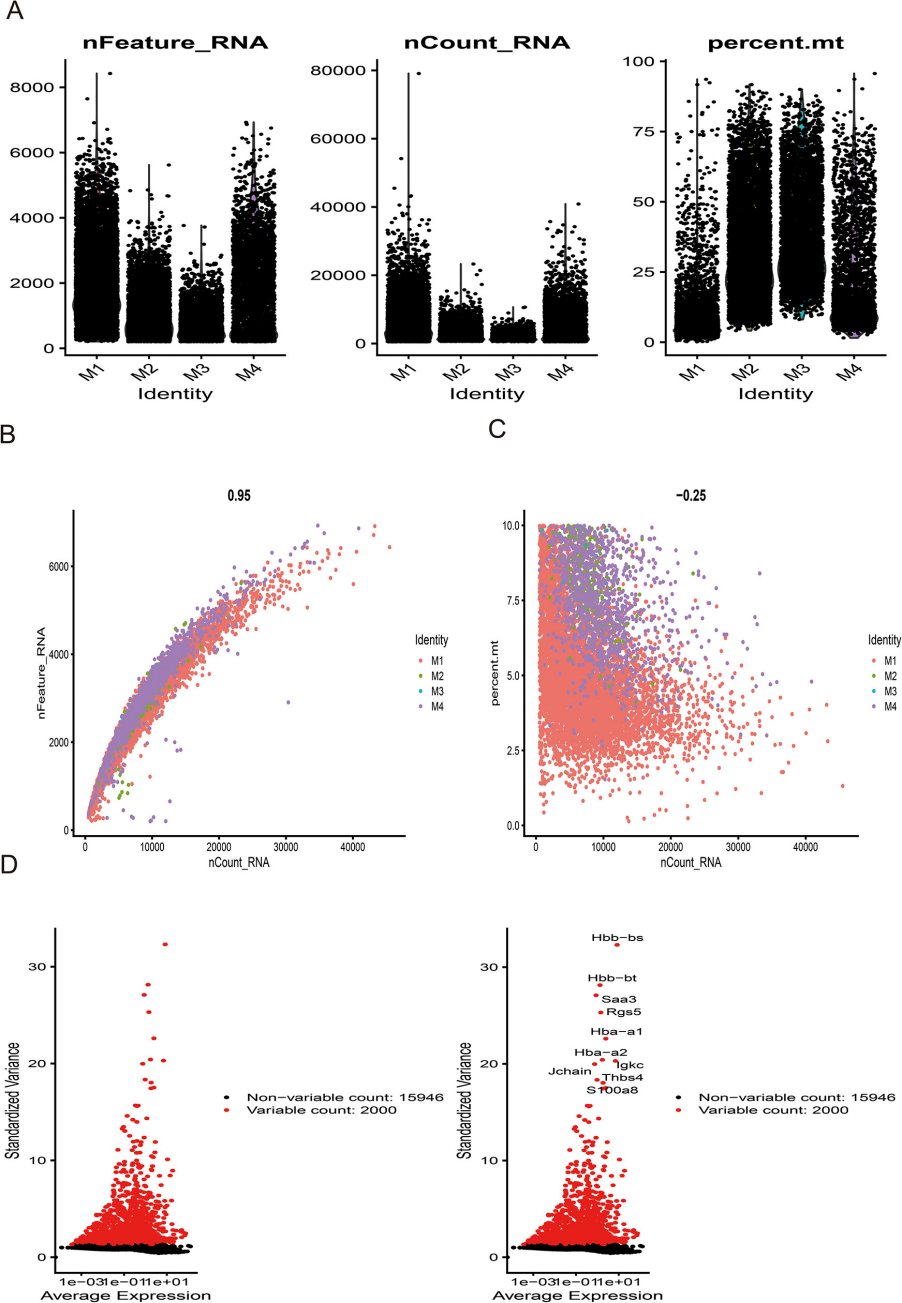
Sample quality control and cell annotation. **(A)** Violin plots depict quality metrics: gene counts per cell (nCandidate_RNA), UMIs per cell (nCount_RNA), and mitochondrial gene percentage (percent.mt). **(B,C)** Scatterplots illustrates various quality control modes. **(D)** Variance plot highlights 1,500 highly variable genes (red dots).

**Figure 8:**
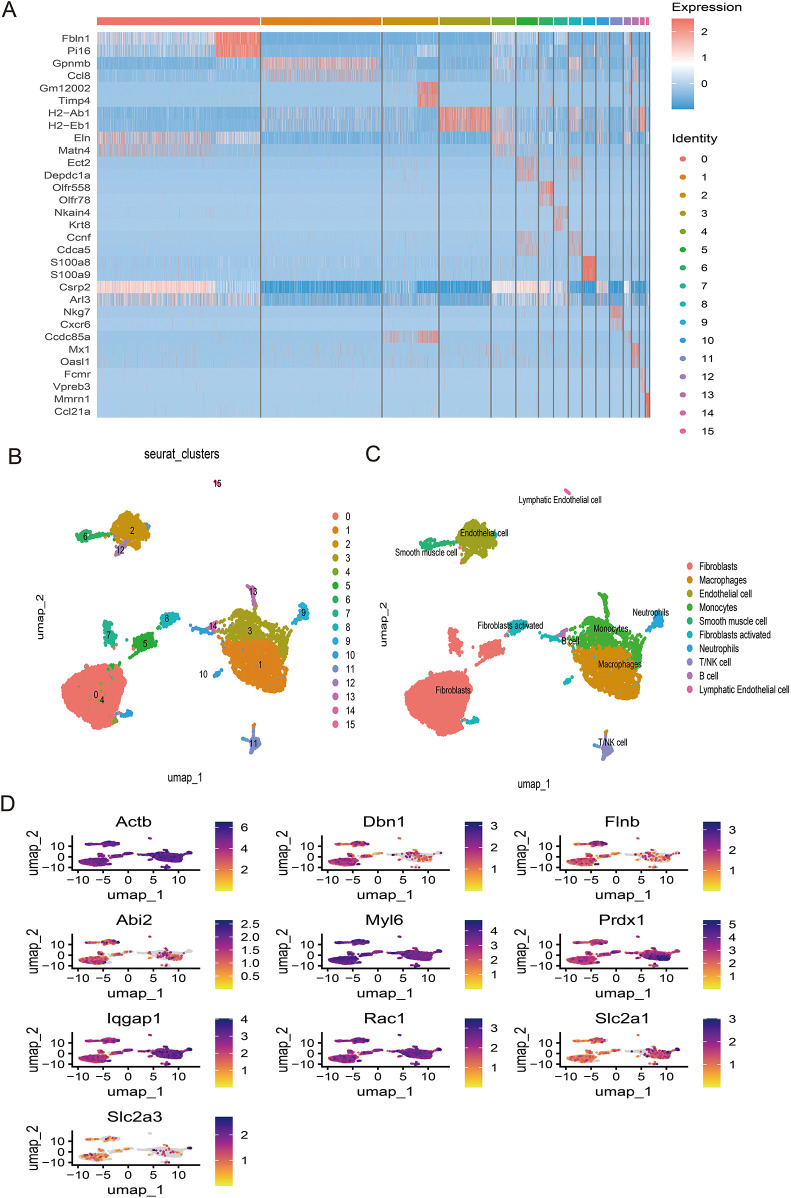
**(A)** Dimheatmap visualizes feature expression patterns. **(B)** Umap-clustering results are presented. **(C)** Ten cell types were annotated. **(D)** The umap plot illustrates the expression of 10 key genes.

**Figure 9:**
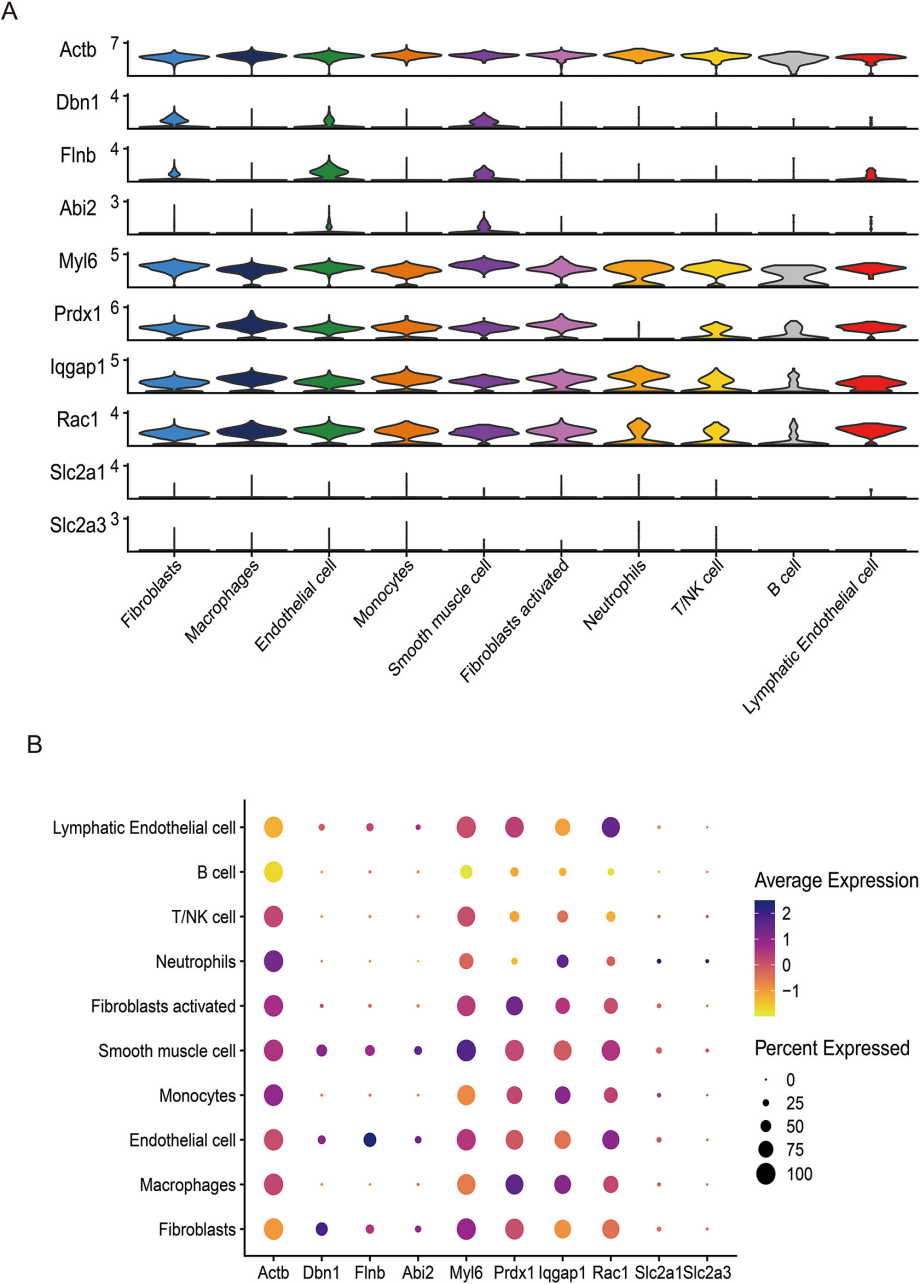
The UMAP plot illustrates the expression of 10 key genes (Actb, Dbn1, Flnb, Abi2, Myl6, Prdx1, Iqgap1, Rac1, Slc2a1, Slc2a3), complemented by violin plots **(A)** and bubble plots **(B)** showing the expression distribution of these genes across different cell types.

Following stringent quality control (Figure 7A), low-quality cells were excluded based on pre-defined thresholds. A strong positive correlation was observed between total RNA counts (nCount) and detected genes (nFeature) (Pearson's r = 0.95, *P* < 0.001; Figure 7B). Subsequent identification of 2,000 highly variable genes enabled downstream analyses (Figure 7D). Dimensionality reduction via principal component analysis (PCA) resolved 16 transcriptionally distinct clusters (Figures 8B), with cluster-specific top 2 marker genes visualized in a heatmap (Figure 8A). Reference-based annotation using the SingleR package classified seven major cell types: smooth muscle cells(SMC),fibroblasts, neutrophils, macrophages, endothelial cell(EC),monocytes, fibroblasts activated, T/NK cell, B cell and Lymphatic Endothelial cell, each color-coded for clarity (Figure 8C). Spatial distribution analysis of 10 key genes revealed ubiquitous expression across multiple lineages (Figure 8D). Spatiotemporal mapping of the 10 key genes across heterogeneous cell populations revealed lineage-specific expression hierarchies (Figure 9). Eight genes exhibited predominant enrichment in smooth muscle cells (SMCs) and endothelial cells (ECs), while Slc2a3 and Slc2a1 diverged from this pattern. The genes Actb, Myl6, Iqgap1, and Rac1 are associated with the expression profiles of neutrophils, macrophages, T/NK cells, and B cells. Additionally, Dbn1 shows a moderate level of expression in fibroblasts, endothelial cells, and smooth muscle cells. Notably, Slc2a3 exhibits some level of expression in neutrophils and smooth muscle cells.

## 4 Discussion

Acute Myocardial Infarction (AMI) is a major public health issue globally, including in China. As an acute coronary syndrome influenced by genetic and environmental factors, AMI is a severe cardiovascular event. Although diagnostic and treatment technologies have advanced, the limited regenerative capacity of cardiomyocytes makes it difficult to repair damaged heart tissue. Post-AMI, extensive cardiomyocyte death leads to cardiac dysfunction and accelerates the progression to heart failure (5). Maintaining a balance between cardiomyocyte survival and death is crucial for preventing AMI and protecting cardiac function. Disulfidptosis is a novel form of cell death. While there are no related studies on its role during acute myocardial infarction, given the various forms of cardiomyocyte death involved in AMI, Disulfidptosis may play a role in the pathogenesis of acute myocardial infarction.

This study identified ten key genes (ACTB, RAC1, IQGAP1, FLNB, MYL6, ABI2, DBN1, PRDX1, SLC2A1, and SLC2A3) implicated in acute myocardial infarction through various bioinformatics approaches. Their diagnostic value was corroborated across the external dataset GSE61144. Elevated expression levels of RAC1, IQGAP1, MYL6, DBN1, SLC2A1 and SLC2A3 were observed in acute myocardial infarction patients compared to healthy controls, suggesting a strong association with disease progression and indicating their potential as novel therapeutic targets.

Our protein-protein interaction (PPI) network analysis revealed that the hub genes DBN1 and SLC2A3 lack direct connectivity but are indirectly linked through ACTB, which serves as a critical mediator (Figures 4A,B). The ACTB gene encodes β-actin, a highly abundant and conserved cytoskeletal structural protein. β-actin facilitates cell migration, division, growth, signaling, and cytoskeletal organization, underscoring its essential role in maintaining cellular morphology (41, 42). Notably, emerging evidence associates ACTB-encoded β-actin with vascular remodeling—a recognized risk factor for cardiovascular diseases (43). A prior study further implicated ACTB in acute myocardial infarction (AMI) susceptibility, though the precise mechanistic basis remains undefined (44). Collectively, these findings suggest that ACTB may orchestrate synergistic effects of DBN1 and SLC2A3 in AMI pathogenesis, potentially through complex cytoskeletal regulatory cascades warranting further investigation.

Building upon these insights, we performed drug-target prediction analyses (Table 3), identifying quercetin (CTD ID: 00006679) as a multi-target agent capable of modulating seven disulfidptosis-related genes, including PRDX1, SLC2A1, FLNB, SLC2A3, IQGAP1, ACTB, and DBN1. This polypharmacological profile suggests quercetin's therapeutic potential in AMI, likely through multi-target modulation of cytoskeletal and redox homeostasis pathways.Quercetin is a well-documented antioxidant that acts through multiple mechanisms, including the inhibition of xanthine oxidase, NADPH oxidase, and the Fenton reaction, all of which reduce reactive oxygen species (ROS) production. Studies have shown that quercetin can alleviate myocardial inflammation and apoptosis both *in vitro* and *in vivo*. In a left coronary artery ligation-induced I/R injury model, oral administration of quercetin at 2, 10, or 20 mg/kg for five consecutive days significantly reduced serum and myocardial levels of TNF-α, IL-6, and IL-1β. Decreased levels of CK and LDH were also observed, along with a notable reduction in myocardial infarct size.Evidence suggests that these protective effects may involve the HMGB1 signaling pathway, which plays a central role in early inflammatory responses. In murine I/R models, quercetin treatment downregulated the expression of pro-inflammatory cytokines compared to untreated controls. The anti-inflammatory and anti-apoptotic effects of quercetin appear to be mediated, at least in part, through regulation of the HMGB1/TLR4/NF-*κ*B signaling pathway (45). Furthermore, we developed a diagnostic nomogram integrating the two hub genes (DBN1 and SLC2A3) (Figure 6). The model demonstrates that elevated expression levels of both genes correlate with increased AMI probability, supporting their utility as a combinatorial biomarker panel. In the future, toward a Multi-Omics Diagnostic Framework, advocating the integration of DBN1 (structural regulation), RAC1 (oxidative signaling), and SLC2A3 (metabolic stress) into a machine learning-driven diagnostic panel.

DBN1 and SLC2A3 are the final hub genes identified in our analysis, and they show potential as candidate biomarkers for the early diagnosis of acute myocardial infarction. DBN1 encodes the actin-binding protein drebrin1 (46). Takanori et al. isolated cardiac fibroblasts from a mouse model of myocardial infarction three days after the event. They found that DBN1 gene knockout was associated with altered protein levels of *α*-SMA and SMA22*α* in these fibroblasts. Further studies on the impact of drebrin on actin cytoskeleton formation indicated that DBN1 gene knockout significantly inhibits the formation of the F-actin cytoskeleton, demonstrating that drebrin appears to facilitate actin cytoskeleton formation in cardiac fibroblasts. Additionally, drebrin was correlated with enhanced actin–MRTF–SRF signaling pathway activity, which coincides with cardiac fibroblast differentiation and increases the expression of the fibrinogen gene Ctrc1, thereby contributing to cardiac fibrosis (47). Furthermore, research in mouse models has shown that the F-actin-binding protein drebrin expression shows an inverse association with atherosclerotic progression. This effect is possibly due to the downregulation of KLF4, Nox1, and ROS levels in smooth muscle cells, which reduces their transdifferentiation into foam cells. Drebrin may also decrease the incidence of macrophages producing smooth muscle cells in atherosclerosis, thereby mitigating the condition (48). Therefore, DBN1 expression patterns suggest potential involvement in AMI pathophysiology through associations with vascular atherosclerosis and cardiac fibrosis.

SLC2A3 encodes GLUT3, facilitating glucose transport for cellular energy metabolism, and is expressed in human heart cells (49). Hypoxia boosts HIF-1, upregulating SLC2A3 and glucose use (50). In acute myocardial infarction, involving hypoxia and energy disruption, SLC2A3 expression correlates with metabolic changes during AMI. Though no direct links prove SLC2A3's role in AMI prognosis, GLUT overexpression has been observed alongside improved outcomes in murine ischemia models (51). Bioinformatics and machine learning associate SLC2A3 with potential biomarker characteristics for AMI detection (52, 53), regulated by the MAPK pathway. Our findings demonstrate SLC2A3 upregulation concurrent with AMI presentation, indicating its importance.

Our study identifies 10 genes potentially linked to disulfidptosis, yet their functional significance in acute myocardial infarction (AMI) requires further investigation. While no direct evidence currently connects disulfidptosis with other regulated cell death pathways in AMI, we hypothesize plausible interactions through shared pathological mechanisms. Oxidative stress may serve as a potential convergence point, where glutathione depletion coupled with SLC7A11 impairment could simultaneously promote disulfidptosis (via cytoskeletal disulfide accumulation) and ferroptosis (through iron-mediated lipid peroxidation) during ischemia-reperfusion injury (35, 54). Inflammatory crosstalk might further interconnect these pathways: cytoskeletal disruption from disulfidptosis could release damage-associated molecular patterns (DAMPs), potentially enhancing RIPK1/RIPK3-MLKL-dependent necroptosis, while inflammatory mediators from necroptosis may indirectly aggravate disulfidptosis by compromising thioredoxin-mediated disulfide resolution (55). Spatial heterogeneity in metabolic status may also influence pathway dominance, with apoptosis potentially prevailing in ATP-sufficient peri-infarct zones and disulfidptosis becoming prominent in ATP-depleted ischemic cores (56). Planned single-cell investigations aim to delineate the spatiotemporal expression patterns of these genes during AMI progression. This theoretical framework, anchored by shared regulatory nodes like SLC7A11 and redox-sensitive mechanisms, suggests opportunities for exploring multitarget therapeutic approaches. By proposing these speculative interactions, our study highlights the complexity of cell death regulation in ischemic injury and underscores the necessity for systematic experimental validation to clarify pathway interplay in distinct myocardial microenvironments.

Single-cell sequencing analysis identified 10 key genes exhibiting predominant expression in endothelial cells and smooth muscle cells (SMCs), suggesting their potential regulatory roles in AMI pathogenesis through cellular-specific mechanisms. Emerging evidence highlights the critical involvement of endothelial cells in post-infarction cardiac repair. Following myocardial injury, endothelial cells facilitate tissue repair through proliferative activity and angiogenic sprouting to establish neovascular networks. Notably, activation of the canonical Wnt signaling pathway triggers endothelial-to-mesenchymal transition, generating myofibroblast-like cells that may contribute to fibrotic remodeling (57). Lehanna et al. demonstrated that Grem2 attenuates inflammatory responses and enhances cardiac functional recovery through inhibition of canonical BMP signaling, which modulates inflammatory cell infiltration in post-MI myocardium. Mechanistically, BMP pathway activation in endothelial cells promotes monocyte adhesion, positioning these cells as primary mediators of Grem2's anti-inflammatory effects during cardiac repair (58). These collective findings underscore endothelial cells as promising therapeutic targets for post-infarction myocardial regeneration.

Regarding vascular smooth muscle cells, accumulating evidence establishes their pivotal role in arterial remodeling following vascular injury and potential utility in post-AMI functional recovery. Experimental studies reveal NF-*κ*B's essential role in SMC activation and proliferation *in vitro*, as well as atherosclerotic plaque formation *in vivo*. The zinc finger protein A20, a potent negative regulator of NF-*κ*B signaling, effectively suppresses pro-inflammatory responses and atherogenic processes in SMCs by inhibiting NF-*κ*B activation, thereby attenuating neointimal hyperplasia (59). These findings not only elucidate the anti-inflammatory capacity of A20 in vascular SMCs but also highlight their therapeutic potential for mitigating pathological vascular remodeling. Consequently, SMCs emerge as another viable cellular target for developing novel therapeutic strategies against AMI progression.

This research relies on transcriptome data from publicly accessible databases, which introduces some limitations.Limitations include small cohorts.Despite cross-validation and bootstrap mitigations, larger validation cohorts are required to confirm the two-gene signature's generalizability.To confirm the diagnostic significance of these biomarkers, it is crucial to gather blood samples from diverse populations for testing. Additionally, creating cell and animal models is essential for a more in-depth exploration of the mechanisms underlying ferroptosis in acute myocardial infarction (AMI). It should be noted that this study did not stratify gene expression profiles by sex. Sex-based differences in clinical presentation, pathophysiology, and outcomes of acute myocardial infarction are well recognized. Future studies with larger cohorts are warranted to explore sex-specific biomarkers and regulatory mechanisms through stratified analyses.Our study identifies critical limitations requiring experimental validation:myocardial expression patterns of disulfidptosis-associated genes (DBN1/SLC2A3) under ischemia need verification through qPCR, immunoblotting, and spatial transcriptomics in cardiac tissues.Functional roles in redox-mediated cytoskeletal remodeling require testing using hypoxia/reoxygenation models with primary cardiomyocytes or engineered heart tissues. Future investigations should integrate multi-omics profiling and CRISPR-based functional analyses to validate these computational predictions mechanistically. The study utilized public gene datasets lacking detailed clinical metadata, potentially introducing confounders. Despite normalization efforts, future research in well-annotated cohorts is needed to validate findings and address these limitations.

## 5 Conclusion

Through bioinformatics analysis, we identified two hub genes associated with disulfidptosis (SLC2A3 and DBN1) that exhibit significant diagnostic potential. These genes appear to be closely linked to the mechanism of acute myocardial infarction.Targeting disulfidptosis could offer a novel therapeutic approach for managing acute myocardial infarction.

## Data Availability

Our study utilized the following publicly available datasets from the Gene Expression Omnibus (GEO) database related to acute myocardial infarction: GSE60993, GSE61144, GSE163956.
