# Supplementary figures and images for: Disulfidptosis-related gene in acute myocardial infarction and its diagnostic value and functions based on bioinformatics analysis and machine learning
Source: Front Cardiovasc Med. 2025 Jul 2;12:1513342. doi: 10.3389/fcvm.2025.1513342 (PMC12263613; doi:10.3389/fcvm.2025.1513342)

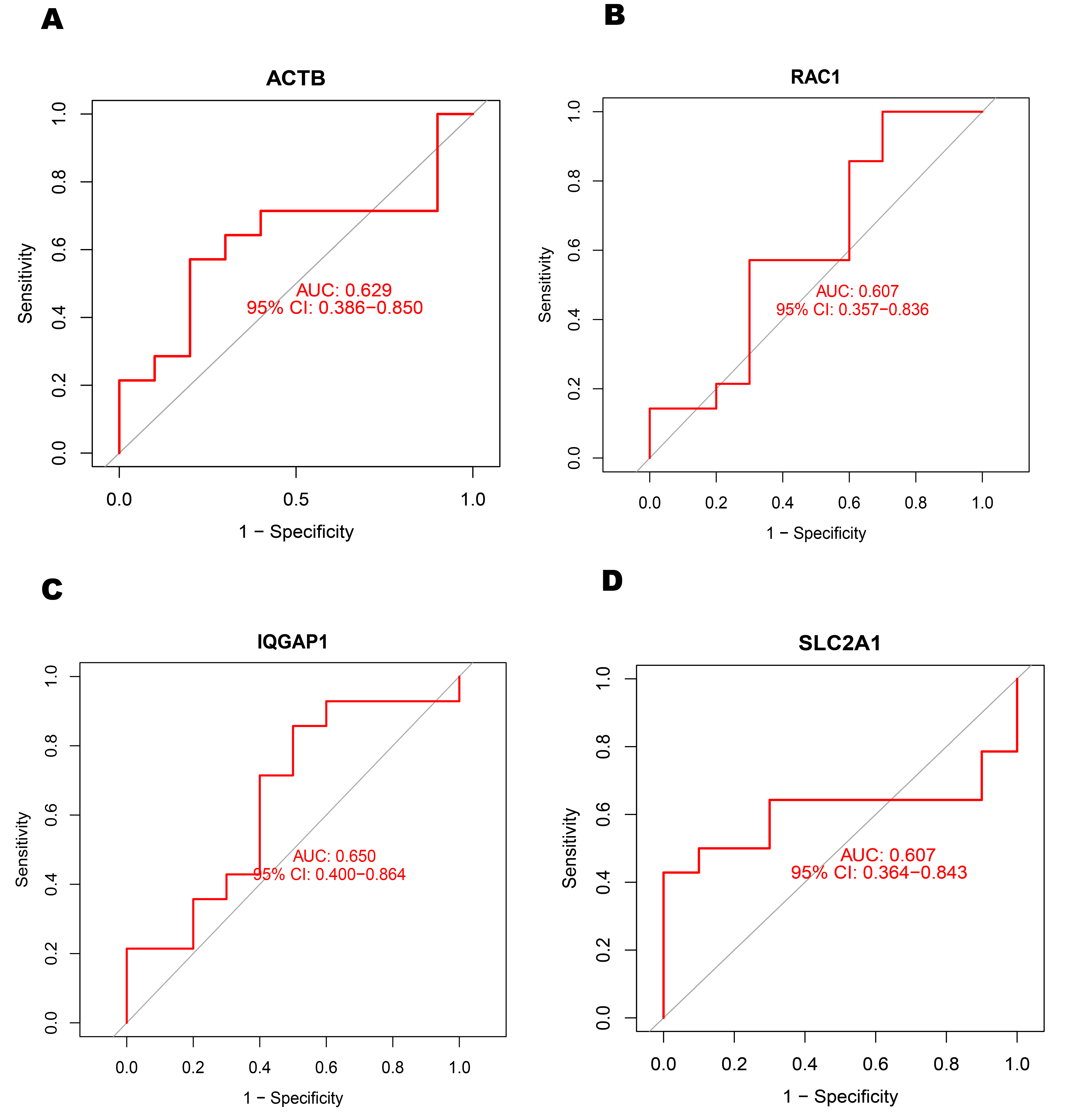

Supplement: Supplementary file 1 [file Image1.jpeg]
